# Anthraquinonyl glycoside facilitates the standardization of graphene electrodes for the impedance detection of lectins

**DOI:** 10.1186/s13065-014-0067-y

**Published:** 2014-11-25

**Authors:** Bi-Wen Zhu, Liang Cai, Xiao-Peng He, Guo-Rong Chen, Yi-Tao Long

**Affiliations:** Key Laboratory for Advanced Materials & Institute of Fine Chemicals, East China University of Science and Technology, 130 Meilong Rd, Shanghai, 200237 PR China

**Keywords:** Anthraquinone, Graphene, Glycoside, Lectin, Electrochemistry, EIS, Standardization

## Abstract

**Background:**

Construction of electrochemical impedance sensors by the self-assembly technique has become a promising strategy for the ‘label-free’ detection of protein-ligand interactions. However, previous impedance sensors are devoid of an *inherent* electrochemical signal, which limits the standardization of the sensors for protein recognition in a reproducible manner.

**Results:**

We designed and synthesized an anthraquinonyl glycoside (AG) where the anthraquinone (AQ) moiety can bind to the surface of a graphene-based working electrode while the glycoside serving as a ligand for lectin. By measuring the inherent voltammetric signal of AQ, the glycosides decorated on the working electrode could be simply quantified to obtain electrodes with a unified signal window. Subsequently, impedance analysis showed that the ‘standardized’ electrodes gave a reproducible electrochemical response to a selective lectin with no signal variation in the presence of unselective proteins.

**Conclusion:**

Anthraquinone-modified ligands could be used to facilitate the standardization of electrochemical impedance sensors for the reproducible, selective analysis of ligand-protein interactions.

**Electronic supplementary material:**

The online version of this article (doi:10.1186/s13065-014-0067-y) contains supplementary material, which is available to authorized users.

## Background

Sugars distributed on the surface of mammalian cells are key informational molecules for cell-cell recognition and adhesion through the interaction with lectins (sugar recognition proteins). Unquestionably the ability to probe sugar-lectin recognitions may boost the advancement of the glycomics. However, conventional approaches for analysis of these interactions mainly rely on immunofluorescence techniques, which are time-consuming and expensive. As a result, a number of ‘label-free’ methods for the quick and economic detection of lectins have been developed [1-5].

Among the various methods introduced, electrochemistry, because of its ease in manipulation and good sensitivity, has been widely employed for lectin analyses [3,5,6]. In addition, electrochemical techniques generally do not require heavy facilities for signal output. Electrochemical impedance spectroscopy (EIS) can sensitively interpret the resistive ability of an interfacial species, which has been broadly applied in the study of corrosion science as well as development of label-free sensors. EIS sensors for lectins, based on the gold-alkenethiol self-assembly technique, have provided promising means for the concise, label-free detection of lectins and live cells that express a glyco-receptor [7-15].

Nevertheless, while the use of gold as working electrode may increase the detection cost, the standardization of electrodes remains difficult due to the lack of an inherent signal ‘reporter’. To address these issues, we report here the design and synthesis of an anthraquinonyl glycoside (AG) in which the anthraquinone moiety can simultaneously serve as a ‘binder’ for a graphene-based electrode and a reporter that produces an electrochemical signal to standardize the sensor fabrication. By using voltammetry, the AGs decorated on the graphene-based electrodes can be easily quantified, thereby facilitating the standardization of the electrodes to produce a unified signal window for lectin detection. Subsequently, EIS analyses showed that the standardized electrodes gave a highly reproducible electrochemical response to a selective lectin, suggesting the promise of using anthraquinone-modified glyco-ligands for the impedance detection of lectins.

## Results and discussion

**Scheme 1:**
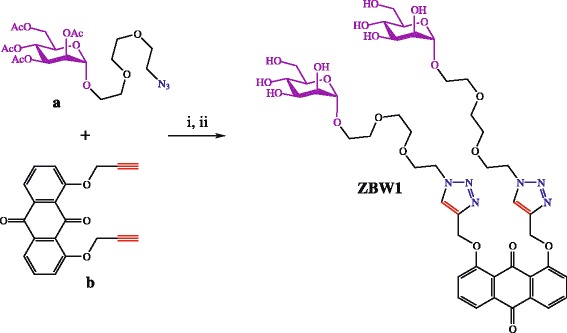
**Synthesis of the target anthraquinonyl mannoside.** Reagents and condition: **(i)** CuSO_4_ · H_2_O, Na ascorbate, CH_2_Cl_2_/H_2_O; **(ii)** Et_3_N, MeOH/H_2_O.

As shown in Scheme 1, the desired anthraquinonyl glycoside (**ZBW1**) was synthesized by the Cu(I)-catalyzed azide-alkyne 1,3-dipolar cycloaddition (CuAAC) of azido mannoside **a** [16] with dipropargyl anthraquinone **b** [5], followed by a de-acetylation, in 66% yield. For the sensor fabrication, the compound was simply spotted to the working electrode (pre-coated with a nano-graphene (**nG**)) of a screen printed electrode [17]. The presence of graphene may increase the adsorption of AG onto the working electrode [18]. Comparing to the conventional gold-thiol self-assembly, the strong π-interaction between graphene and anthraquinone [5] may provide a more facile and economic means for construction of self-assembled electrochemical biosensors due to the preclusion of using gold as the sensing platform. Upon formation of the sensors, the hydrophilic glycosyl moiety could expose to the environment for lectin recognition [5].

**Figure 1:**
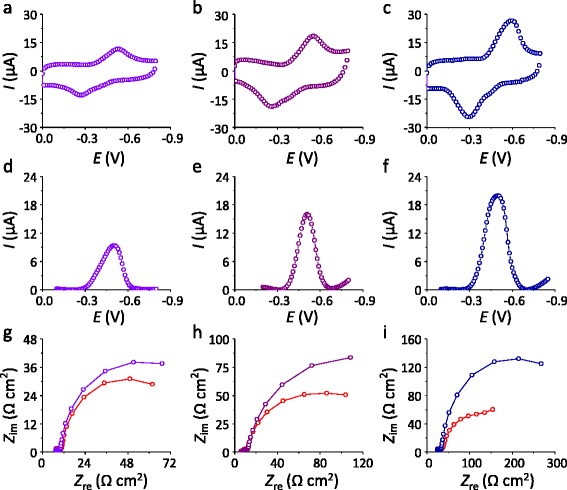
**Representative electrochemical methods for the standardized detection of mannose-Con A interactions.** Cyclic voltammetry (CV) of **ZBW1** with a surface converge (*Г*) of **(a)** 1.1 × 10^−9^ mol cm^−2^, **(b)** 2.7 × 10^−9^ mol cm^−2^ and **(c)** 6.9 × 10^−9^ mol cm^−2^; Differential pulse voltammetry (DPV) of **ZBW1** with a current intensity of **(d)** 9.3 μA, **(e)** 15.2 μA and **(f)** 19.9 μA; Electrochemical impedance spectroscopy (EIS) of **ZBW1** with a lectin coverage efficiency (*η*) of **(g)** 34.8%, **(h)** 49.7%, and **(i)** 72.5% on graphene electrodes in the presence (colored) of Con A (10 μM) (red plots stand for **ZBW1**-functionalized graphene electrodes in the absence of a lectin).

Voltammetry and EIS were used to monitor the sensor standardization. Three sets of electrodes with increasing current intensities (Figure 1d: 9.3 μA, Figure 1e: 15.2 μA and Figure 1f: 19.9 μA) were made by spotting **ZBW1** with different concentrations to the working electrode. The surface coverage areas (*Г*, adsorbed AQ species) of the different electrodes were determined to be 1.1 × 10^−9^ (Figure 1a), 2.7 × 10^−9^ (Figure 1b) and 6.9 × 10^−9^ (Figure 1c) mol cm^−2^ by cyclic voltammetry [3,5]. EIS was then used to analyze the surface adhesion of a mannose-selective lectin, Concanavalin A (Con A), using [Fe(CN)_6_]^3-/4-^ as a redox probe [5]. We observed that the charge transfer resistance (*R*_ct_) of all sets of electrodes decorated with the mannoside increased evidently in the presence of Con A (Figure 1g-i), suggesting the adhesion of the lectin onto the electrode surface. This is in good agreement with previous observations [5,11].

The lectin coverage efficiency (*η*) was used to interpret the recognition, which was calculated by the following equation [19]:

1 $$ \eta =\left[{R}_{\mathrm{ct}}-{R}_{\mathrm{ct}(0)}\right]/{R}_{\mathrm{ct}} $$

where *R*_ct_ and *R*_ct(0)_ are the charge transfer resistance in the presence and absence of lectin, respectively.

**Figure 2:**
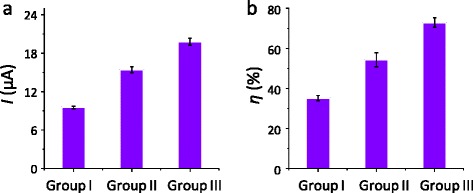
**Standardized detection of mannose-Con A interactions via electrochemical impedance spectroscopy. (a)** Averaged current density (*I*) of **ZBW1** decorated on the graphene electrode of different groups. **(b)** Averaged lectin coverage efficiency (*η*) of the graphene electrode decorated with **ZBW1** of different groups in the presence of Con A (10 μM). The mean *I* for group I, II and II are 9.6 ± 0.2, 15.4 ± 0.5 and 19.8 ± 0.6 μA, respectively. The mean *η* for group I, II and II are 35.2 ± 1.3, 54.3 ± 3.5 and 72.9 ± 2.4%, respectively. The original DPV and EIS plots of group I, II and III are shown in Additional file 1: Figures S1, S2 and S3, respectively.

Notably, the electrodes with increasing current densities showed, consistently, increasing *R*_ct_ upon addition of Con A (Figure 1g: 34.8%, Figure 1h: 49.7% and Figure 1i: 72.5%). This implies that the current intensity of AQ, which is related to the total amount of molecules adsorbed onto the electrode, corresponds well with the subsequent impedance change of the glyco-electrode in the presence of lectin. As a result, we produced series of electrodes (five for each set) with increasing current intensities of 9.6 ± 0.2, 15.4 ± 0.5 and 19.8 ± 0.6 μA (Figure 2a). The *η* of the electrodes were determined to be 35.2 ± 1.3, 54.3 ± 3.5 and 72.9 ± 2.4%, respectively (Figure 2b). These data further suggest the following facts: 1) The electrodes with nearly identical current intensities show reproducible EIS response to a selective lectin, and 2) AQ, while conjugated with a biomolecule, could be exploited as a signal reporter to standardize the EIS-based biosensor fabrication via voltammetry.

**Figure 3:**
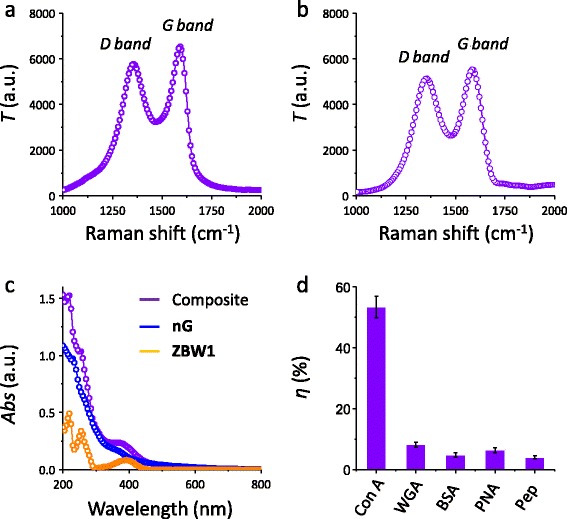
**Characterization of the compound-graphene complex and sensor selectivity.** Raman spectra of **(a)** nano-graphene (**nG**) and **(b) nG** complexed with **ZBW1**; **(c)** UV–vis spectra of **nG**, **ZBW1** and the complex; **(d)** Selectivity of **ZBW1**- decorated graphene electrode for different proteins including the *N*-acetyl glucosamine-selective wheat germ agglutinin (WGA), the galactose-selective peanut agglutinin (PNA), bovine serum albumin (BSA) and pepsin (Pep) determined by electrochemical impedance spectroscopy. The original EIS spectra are shown in Additional file 1: Figure S5.

The complexation between AG and graphene was also characterized by various techniques. In the Raman spectra, the intensity ratio of the D band (1355 cm^−1^) to the G band (1600 cm^−1^) of the **ZBW1**-**nG** complex increased (0.92, Figure 3a) comparing to the bare **nG** (0.86, Figure 3b). This suggests an increase in sp^2^-hybridization of the complex probably because of the stacking of the aromatic **ZBW1** to the surface of graphene [4,5,20,21]. In the meanwhile, peaks characteristic of the stacking of AG to **nG** were observed in the UV–vis (red shift from 388 nm [orange] to 395 nm [violet] Figure 3c) and FTIR (*ν̃* = 2350 cm^−1^, Additional file 1: Figure S4) spectra of the **ZBW1**-**nG** complex. These results suggest the successful assembly of the AG-graphene complex. Furthermore, we observed that the CV and DPV of unmodified anthraquinone on the graphene electrode (Additional file 1: Figure S6) are in good agreement with those of **ZBW1**, suggesting the functionalization of the electrodes with the anthraquinone group.

We then tested the selectivity of the biosenor with a series of unselective lectins and proteins including the *N*-acetyl glucosamine-selective wheat germ agglutinin (WGA), the galactose-selective peanut agglutinin (PNA), bovine serum albumin (BSA) and pepsin (Pep). As shown in Figure 3d, the electrode only showed an impedance change in the presence of the selective Con A with no insignificant response to the unselective proteins. This suggests the usefulness of the EIS sensor developed for the selective detection of lectins.

### Experimental section

#### General

All purchased chemicals and reagents are of analytical grade. Nano-graphene (lateral diameters ranging from 1–10 nm) was purchased from Nanjing XFNano Materials Tech. Co., Ltd. Solvents were purified by standard procedures. Reactions were monitored by TLC (thin-layer chromatography) using E-Merck aluminum precoated plates of Silica Gel. ^1^H NMR spectrum was recorded on a Bruker AM-400 spectrometer using tetramethylsilane (TMS) as the internal standard (chemical shifts in parts per million). High resolution mass spectrum was recorded on a Waters LCT Premier XE spectrometer using standard conditions (ESI, 70 eV). High performance liquid chromatogram (HPLC) was taken on an Agilent 1100 equipment.

#### Synthesis of ZBW1

To a solution of **b** (250 mg, 0.50 mmol) and **a** (68.6 mg, 0.25 mmol) in a solvent mixture of CH_2_Cl_2_ (5 mL) and H_2_O (5 mL) were added CuSO_4_ · 5H_2_O (2.0 equiv.) and Na ascorbate (4.0 equiv.). This mixture was stirred over night and then diluted with CH_2_Cl_2_ and washed with brine. The combined organic layer was dried over MgSO_4_, filtered and concentrated in vacuum to provide a crude product. The product was purified by column chromatography (EtOAc/MeOH = 20:1, v/v) to give an intermediate as a yellow solid (249.4 mg, 0.19 mmol). To a solution of the intermediate in a solvent mixture of MeOH (5 mL) and H_2_O (5 mL) was added excessive Et_3_N. This mixture was stirred at room temperature for 36 h. Then, solvent was removed in vacuum to directly afford **ZBW1** as a yellow solid (148.5 mg, 2-step yield 66.4%). *R*_f_ = 0.48 (EtOAc/MeOH = 1:1, v/v). ^1^H NMR (400 MHz, D_2_O): *δ* 8.02 (s, 2H), 7.25-7.14 (m, 6H), 5.06 (s, 4H), 4.63 (s, 2H), 4.51 (t, *J* = 4.0 Hz, 4H), 3.83 (t, *J* = 4.0 Hz, 4H), 3.75-3.74 (m, 2H), 3.69 (d, *J* = 2.0 Hz, 1H), 3.66 (d, *J* = 2.0 Hz, 1H), 3.62 (d, *J* = 3.6 Hz, 1H), 3.61 (s, 1H), 3.60-3.55 (m, 4H), 3.53 (s, 1H), 3.51 (s, 1H), 3.48 (s, 1H), 3.42 (t, *J* = 3.2 Hz, 6H), 3.36 (s, 8H), 3.32-3.28 (m, 1H). HR-ESI-MS: calcd. for [C_44_H_58_N_6_O_20_ + Na]^+^ 1013.3604, found 1013.3606. HPLC: *t*_R_ = 3.9 min over 17 min of eluent (acetonitrile/H_2_O = 9:1, v/v), purity 96.4%.

#### Cyclic voltammetry (CV)

CVs were recorded with a computer controlled CHI 1211B electrochemical station (Chenhua Co. Ltd., Shanghai, China) between −0.8 V and −0.2 V (vs. Ag/AgCl) at a scan rate of 100 mV/s. The electrolyte (Tris–HCl, 0.01 M, pH 7.3) used was degassed with N_2_ for 20 min before measurements. Screen-printed electrodes (SPEs) were pretreated in a PBS (0.05 M, pH 7.0) containing 0.1 M KCl by applying an anodic potential of 2 V (vs. Ag/AgCl) for 200 s, and were then washed with water three times. The circular area (2 mm in diameter) was used as the working electrode; the reference electrode was printed with 40% AgCl in silver paste, and the auxiliary electrode printed with carbon ink. For functionalization of the SPEs, a drop (4 μL) of aqueous **nG** solution was dripped onto the working electrode and dried under vacuum. Then a drop (4 μL) of the aqueous **ZBW1** solution was dripped onto the working electrode and dried under vacuum. The functionalized SPEs were finally immersed into Tris–HCl solutions for recording the CVs.

#### Differential pulse voltammetry (DPV)

DPVs were recorded with an amplitude of 0.05 V, a pulse width of 0.2 s, a standing time of 2 s, and a scanning range from −0.8 V to −0.2 V in Tris–HCl (0.01 M, pH 7.3). For detection of analytes, a drop (4 μL) of the Tris–HCl solution of **ZBW1** (5 × 10^−3^ M) was dripped onto the GO functionalized working electrode area of SPE, and then incubated for 30 min. Then the electrodes were rinsed with the buffer solution three times, dried at room temperature, and then immersed in degassed buffer for measurement.

#### Electrochemical impedance spectroscopy (EIS)

**Figure 4:**
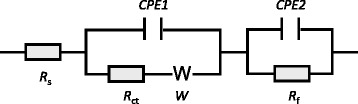
**Circuit model used to fit the Nyquist plots.** *R*_s_ is the solvent resistance, *R*_f_ the film resistance, *R*_ct_ the charge transfer resistance, *CPE1* the film capacitance, *CPE2* the double layer (the functional and electrolyte layer) capacitance, and *W* the Warburg diffusion impedance.

EIS was performed with a ZAHNER apparatus in the presence of the [Fe(CN)_6_]^3−^/[Fe(CN)_6_]^4−^ (5 mM) redox couple in 0.1 M KCl solution in the frequency range of 10 mHz to 100 KHz (perturbation signal: 5 mV). All data collected were fitted with the software ZSimpWin. A drop (4 μL) of the Tris–HCl solution of **ZBW1** (5 × 10^−3^ M) was first dripped onto the graphene functionalized working electrode area of SPE, dried under vacuum, and then the Nyquist plots were recorded. For detection of the sugar-lectin interactions, a drop (Tris–HCl, 4 μL) of protein solution was dripped onto the **ZBW1**-**nG** co-functionalized electrode, dried under vacuum, and the Nyquist plots recorded. Figure 4 shows the circuit model used to fit all Nyquist plots [19]:

#### Fourier Transform Infrared Spectroscopy (FTIR)

FTIR spectra were recorded on a Nicolet 380 FTIR spectrometer (Thermo Electron Corporation, USA). The samples were mixed with KBr and then compressed into pellets for analysis in the spectral range of *ν̃* = 4000 to 500 cm^−1^. All baselines of the spectra were corrected.

#### Raman spectroscopy

Raman spectra were performed on a Renishaw InVia Reflex Raman system (Renishaw plc, Wotton-under-Edge, UK) that employs a grating spectrometer with a Peltier-cooled charge-coupled device (CCD) detector coupled to a confocal microscope, which were then processed with Renishaw WiRE 3.2 software. The Raman scattering was excited by an argon ion laser (*I* = 514.5 nm).

## Conclusion

In summary, we have reported the synthesis of an anthraquinonyl glycoside to fabricate EIS-based electrochemical sensors, where the AQ moiety served as a binder that promotes self-assembly of the glyco-ligands to the working electrode. Importantly, AQ also acted as a signal reporter that facilitates the sensor standardization. By using voltammetry and EIS, we determined that the electrodes with unified current signals showed reproducible impedance response to a selective lectin adhered to the electrode with high selectivity over other unselective proteins. This study provides insights into the simple construction of readily standardizable EIS sensors for the general, economic electrochemical analysis of ligand-protein interactions.

## Additional file

Additional file 1
**Supporting information.**
