# Supplementary material for: Anthraquinonyl glycoside facilitates the standardization of graphene electrodes for the impedance detection of lectins
Source: Chem Cent J. 2014 Nov 25;8:67. doi: 10.1186/s13065-014-0067-y (PMC4245500; doi:10.1186/s13065-014-0067-y)
Supplement: Additional file 1 — Supporting information. [file 13065_2014_67_MOESM1_ESM.pdf]

Supporting Information

Anthraquinonyl glycoside facilitates the standardization of graphene electrodes for the impedance detection of lectins

Bi-Wen Zhu,[1] Liang Cai,[1] Xiao-Peng He,* Guo-Rong Chen and Yi-Tao Long*

Key Laboratory for Advanced Materials & Institute of Fine Chemicals, East China University of Science and Technology, 130 Meilong Rd., Shanghai 200237, PR China

*Corresponding authors:

ytlong@ecust.edu.cn (Y.-T. Long)

xphe@ecust.edu.cn (X.-P. He)

[1]Equal contribution

Contents list:

S1. Additional figures

S1. Additional figures

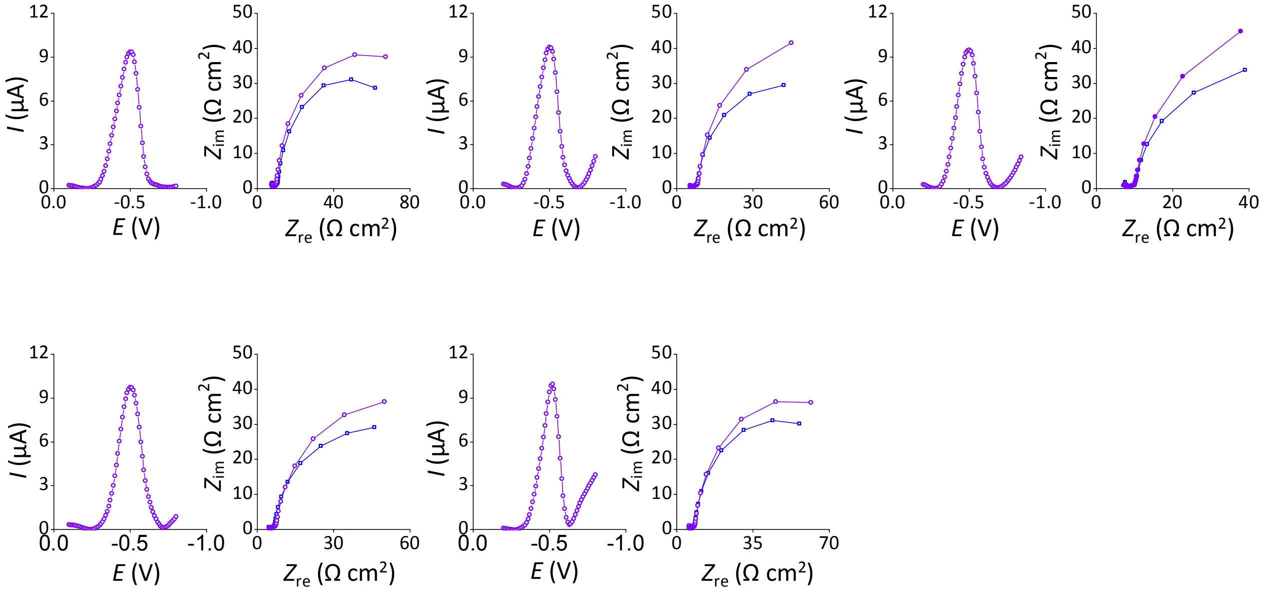

Figure S1. The original DPV and EIS plots of graphene electrodes corresponding to group I of Fig. 2 in the main text.

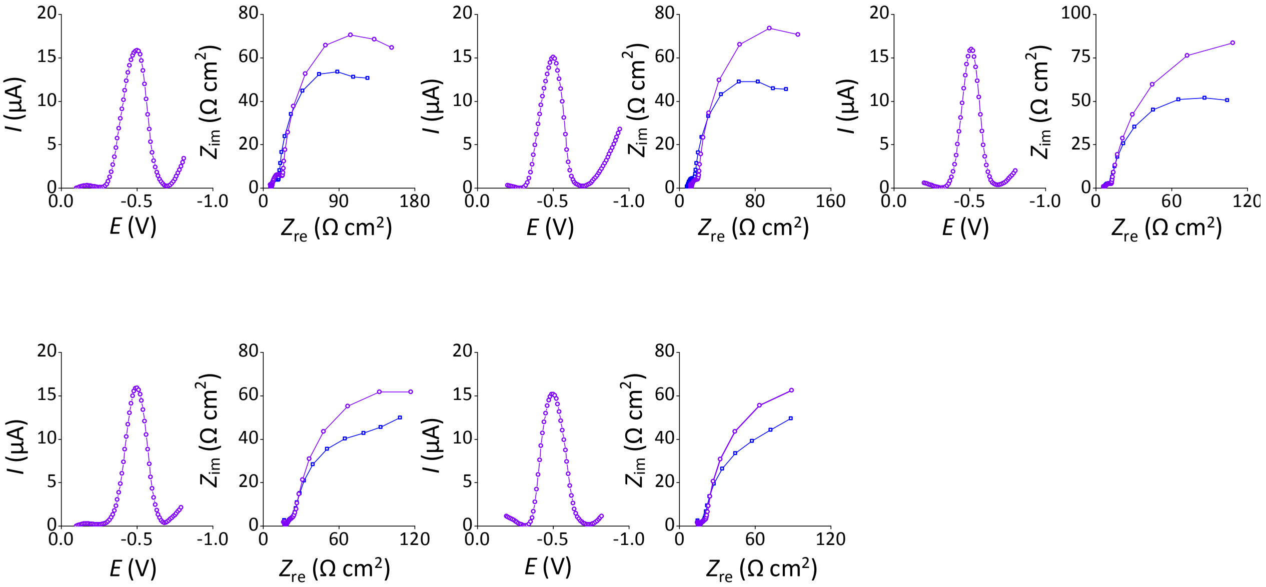

Figure S2. The original DPV and EIS plots of graphene electrodes corresponding to group II of Fig. 2 in the main text.

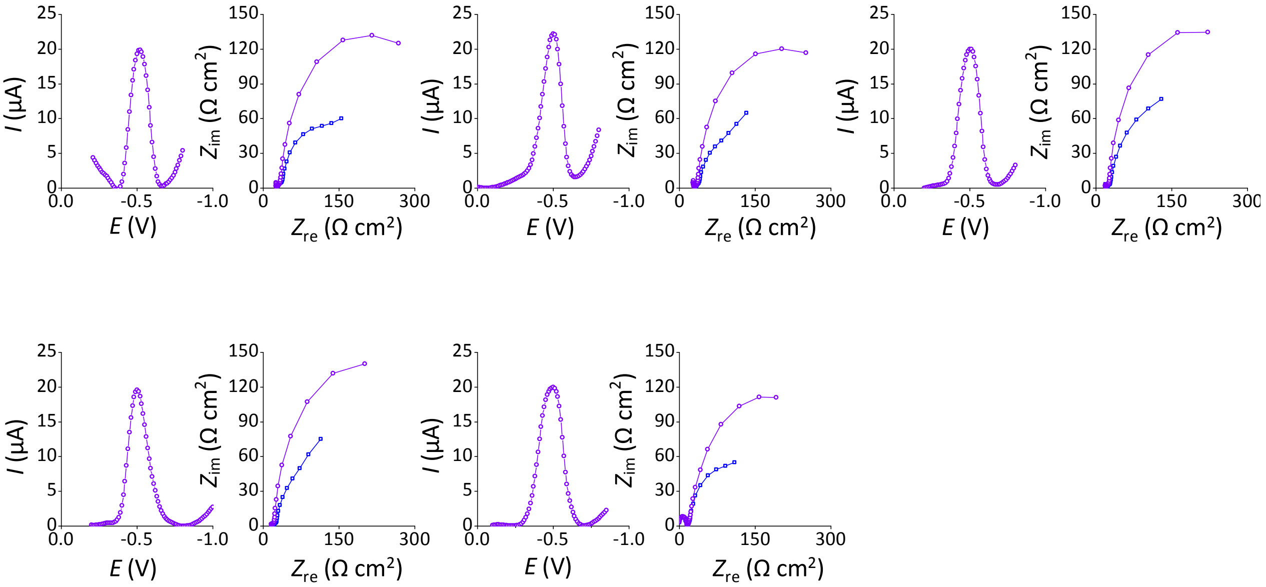

Figure S3. The original DPV and EIS plots of graphene electrodes corresponding to group III of Fig. 2 in the main text.

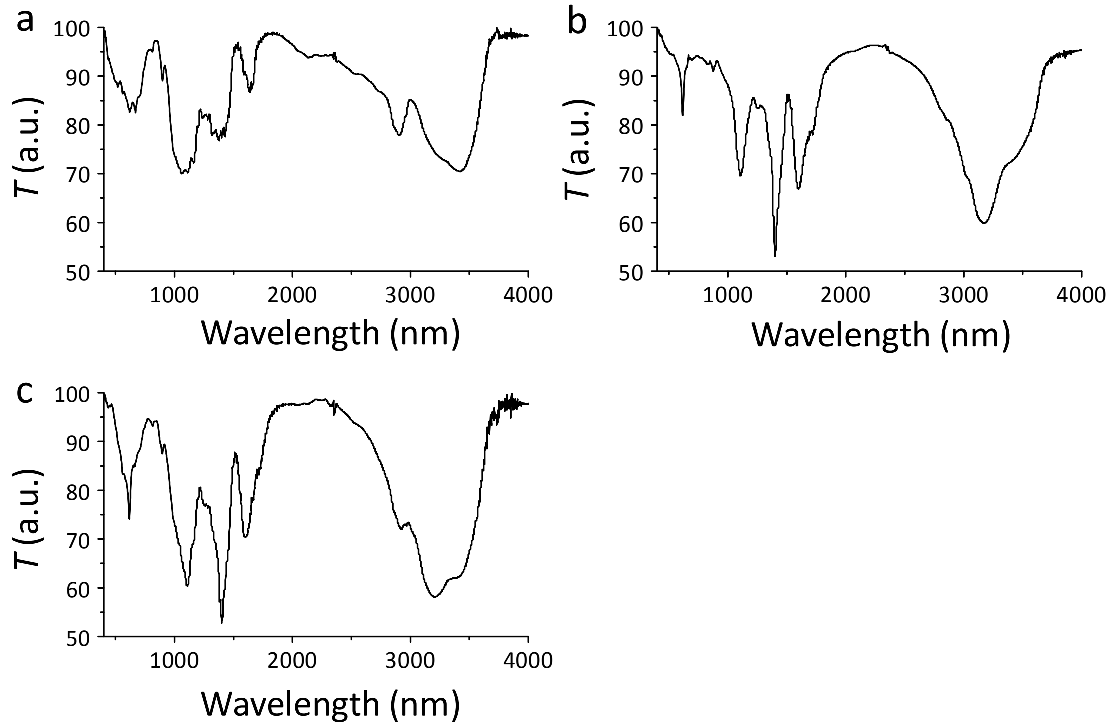

Figure S4. FTIR spectra of (a) nano-graphene (**nG**), (b) **ZBW1** and (c) the **nG**-**ZBW1** complex.

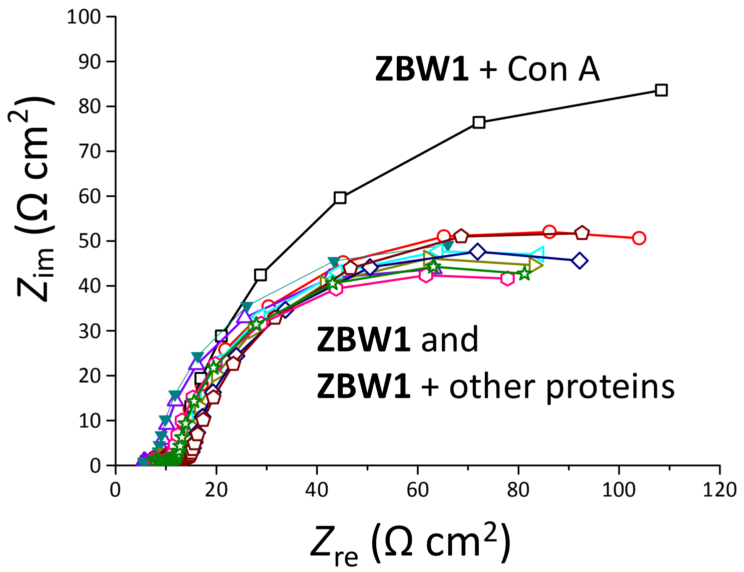

Figure S5. Original EIS spectra of **ZBW1** on graphene electrode in the absence and presence of various proteins (corresponding to Fig. 3d in the main text).

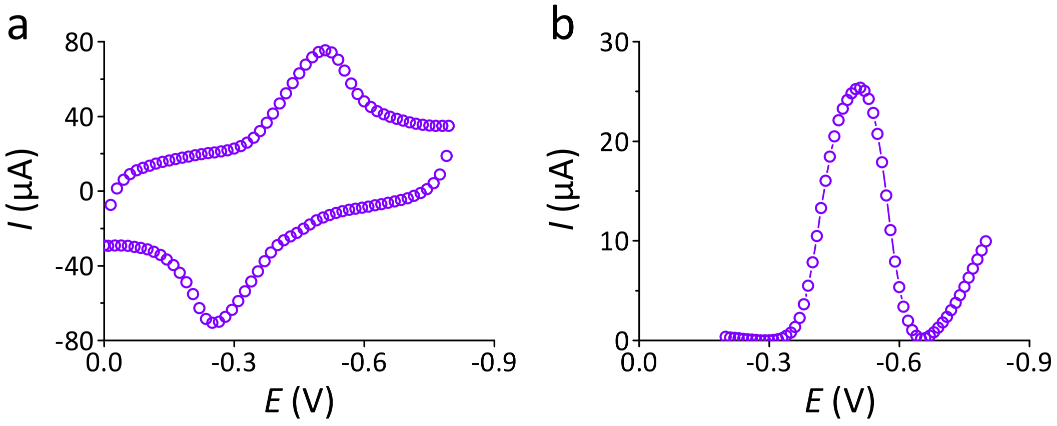

Figure S6. (a) CV and (b) DPV of anthraquinone on the graphene electrode.
